# Impact of clinical context on acute kidney injury biomarker performances: differences between neutrophil gelatinase-associated lipocalin and L-type fatty acid-binding protein

**DOI:** 10.1038/srep33077

**Published:** 2016-09-08

**Authors:** Toshifumi Asada, Rei Isshiki, Naoki Hayase, Maki Sumida, Ryota Inokuchi, Eisei Noiri, Masaomi Nangaku, Naoki Yahagi, Kent Doi

**Affiliations:** 1 Department of Emergency and Critical Care Medicine, The University of Tokyo, Tokyo, Japan; 2 Department of Nephrology and Endocrinology, The University of Tokyo, Tokyo, Japan; 3 Japan Science and Technology Agency/Japan International Cooperation Agency (JST/JICA), Science and Technology Research Partnership for Sustainable Development (SATREPS), Tokyo, Japan

## Abstract

Application of acute kidney injury (AKI) biomarkers with consideration of nonrenal conditions and systemic severity has not been sufficiently determined. Herein, urinary neutrophil gelatinase-associated lipocalin (NGAL), L-type fatty acid-binding protein (L-FABP) and nonrenal disorders, including inflammation, hypoperfusion and liver dysfunction, were evaluated in 249 critically ill patients treated at our intensive care unit. Distinct characteristics of NGAL and L-FABP were revealed using principal component analysis: NGAL showed linear correlations with inflammatory markers (white blood cell count and C-reactive protein), whereas L-FABP showed linear correlations with hypoperfusion and hepatic injury markers (lactate, liver transaminases and bilirubin). We thus developed a new algorithm by combining urinary NGAL and L-FABP with stratification by the Acute Physiology and Chronic Health Evaluation score, presence of sepsis and blood lactate levels to improve their AKI predictive performance, which showed a significantly better area under the receiver operating characteristic curve [AUC-ROC 0.940; 95% confidential interval (CI) 0.793–0.985] than that under NGAL alone (AUC-ROC 0.858, 95% CI 0.741–0.927, *P* = 0.03) or L-FABP alone (AUC-ROC 0.837, 95% CI 0.697–0.920, *P* = 0.007) and indicated that nonrenal conditions and systemic severity should be considered for improved AKI prediction by NGAL and L-FABP as biomarkers.

Acute kidney injury (AKI) is a common and complex condition that is strongly associated with morbidity and mortality in critically ill patients^1^,^2^. Early recognition of AKI is crucial for timely intervention and prevention of complications. Recently, several AKI biomarkers, including neutrophil gelatinase-associated lipocalin (NGAL) and L-type fatty acid-binding protein (L-FABP) have emerged for detection of renal damage earlier than that achieved with serum creatinine. Although several clinical studies evaluating early AKI detection by NGAL and L-FABP showed promising results^3^,^4^, two meta-analyses reported that not all the studies showed area under the receiver operating characteristic curve (AUC-ROC) values of above 0.85, indicating the performance of these biomarkers for early AKI detection could be improved^5^,^6^. Due to the involvement of several distinct pathogenic mechanisms in AKI development, no single biomarker can predict distinct AKI types with equally high sensitivity and specificity; thus, combinational approaches using multiple biomarkers were recommended for improved AKI prediction and detection^7^,^8^,^9^. In addition, information on clinical parameters may improve the performance of AKI biomarkers^10^. However, thus far, few clinical studies have demonstrated the advantage of combining AKI biomarkers with parameters reflecting systemic severity and clinical condition of patients.

Urinary NGAL and L-FABP can increase in response to several distinct renal and nonrenal triggers. NGAL was first found in granules of human neutrophils^11^, followed by several tissues, including the lung, liver and kidney^12^,^13^,^14^,^15^. Renal insults, such as hypoperfusion and interactions with nephrotoxins, upregulate NGAL expression in renal tubular epithelial cells, which is then released into the urine and plasma^16^. NGAL was also recognised as a marker of systemic inflammation^17^, and a strong association between septic AKI and elevated NGAL levels was demonstrated^18^,^19^. Although a small portion of plasma NGAL is filtered by the glomeruli and taken up by proximal tubules via megalin receptors under normal conditions, systemic inflammation precipitates altered reabsorption and stimulates NGAL synthesis in renal and nonrenal tissues, leading to increased urinary NGAL^20^. L-FABP was initially identified in the liver, and hepatic dysfunction was shown to increase serum L-FABP levels^21^,^22^. Furthermore, increased urine L-FABP levels were previously shown to reflect the severity of renal insults^23^. For example, hypoxia induced by hypoperfusion could be detected by changes in urinary L-FABP with high sensitivity even in patients with mild AKI^24^. Reportedly, increased urinary L-FABP levels were observed in non-AKI patients after liver transplantation^25^.

Different mechanisms with some overlaps are evidently involved in increased urinary NGAL and L-FABP levels. Additionally, systemic disturbances, including inflammation, hypoperfusion and liver dysfunction, are important confounding factors in AKI, especially in critically ill patients. However, the impact of these systemic conditions, as well as the disease severity on the efficacy of these AKI biomarkers, has not been extensively investigated. Thus, integrative relationships between NGAL, L-FABP, nonrenal disorders and systemic severity of patients were investigated in the present study to determine whether the performance of these biomarkers for AKI prediction could be improved by including information on clinical context.

## Results

### Patient characteristics

**Figure 1:**
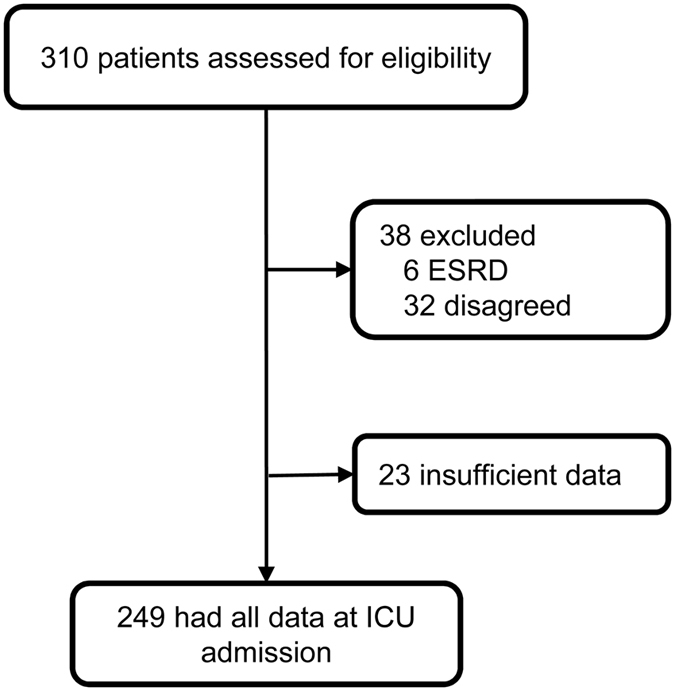
Study flow diagram. ESRD, end-stage renal disease.

A total of 249 patients admitted to our hospital between April 2010 and March 2011 were eligible for analysis (Fig. 1). Of these, 34 patients were diagnosed with chronic kidney disease (CKD) before admission to the intensive care unit (ICU). Additionally, 147 patients (59.0%) were diagnosed with AKI within the first 7 days of ICU admission, 116 of whom had already developed AKI at the time of ICU admission. Fifty-four patients were treated with renal replacement therapy. The in-hospital mortality rate of AKI patients was 19%. Among 147 AKI patients, 10 patients proceeded to CKD, and 9 patients eventually required chronic haemodialysis. Baseline characteristics of the patients with newly developed AKI after ICU admission (n = 31) and those who did not develop AKI during the first 7 days (n = 102) are shown in Table 1.

### Comparison of NGAL and L-FABP in relation to nonrenal disorders

**Figure 2:**
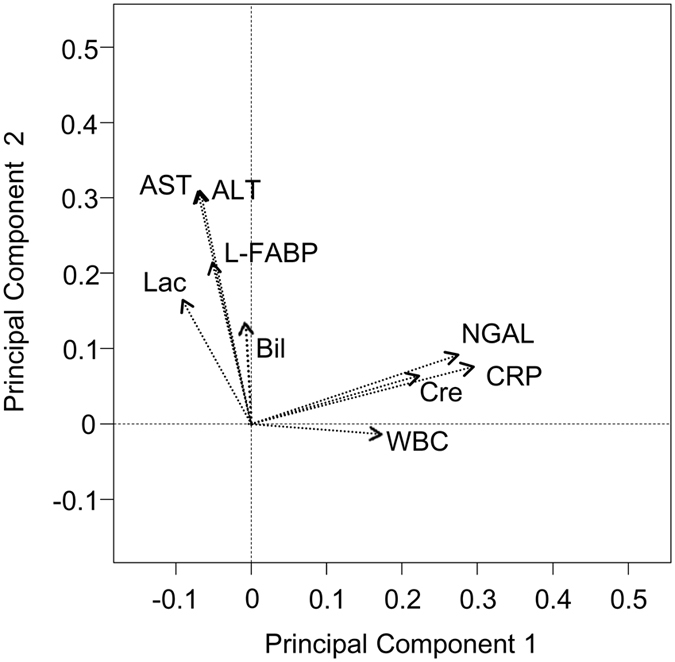
Principal component analysis of acute kidney injury (AKI) biomarkers and systemic parameters. Principal component analysis revealed different orthogonal directions between urinary NGAL and L-FABP.

To examine whether changes in urinary NGAL and L-FABP levels were distinct in relation to nonrenal disorders, we evaluated their relationship with C-reactive protein levels and white blood cell counts for systemic inflammation^26^,^27^, blood lactate levels for hypoperfusion^28^,^29^ as well as aspartate aminotransferase, alanine aminotransferase and total bilirubin levels for hepatic injury^30^,^31^,^32^ using principal component analysis^33^ in all 249 patients. The results of the principal component analysis showed distinct relationships between NGAL and L-FABP and the interrogated variables (Fig. 2). NGAL showed linear correlations with inflammatory indicators and was perpendicular to L-FABP, which was linearly correlated with both hypoperfusion and hepatic injury markers. In addition, the serum creatinine level showed a linear correlation with NGAL. The correlation between NGAL and C-reactive protein level was the strongest, with a correlation coefficient of 0.50 [95% confidence interval (CI) 0.41–0.59]. These distinctive characteristics of NGAL and L-FABP were preserved when AKI and non-AKI patients were separately analysed.

### Impact of hypoperfusion on the predictive ability of NGAL and L-FABP

We compared the predictive performance of urinary NGAL and L-FABP levels by logistic regression analysis using data from 133 patients (31 patients with newly developed AKI within 7 days of ICU admission and 102 patients with no AKI diagnosis throughout the first 7 days of ICU admission). In the absence of patient background information, the AUC-ROCs of NGAL and L-FABP were 0.796 (95% CI 0.697–0.896) and 0.779 (95% CI 0.676–0.883), respectively; no significant differences were observed between their performances (*P* = 0.68).

Based on the result of the principal component analysis which indicated that urinary L-FABP levels might increase in patients with hyperlactatemia independently from renal injury, we first hypothesised that NGAL might be superior to L-FABP as a biomarker in AKI patients with hyperlactatemia. Using a threshold of 2.0 mmol/L for blood lactate level^34^,^35^, 50 patients presenting with hyperlactatemia were identified among the initial 133 patients who were admitted to the ICU without a preexisting AKI diagnosis. Of these, NGAL measured at the time of ICU admission was superior to L-FABP in detecting AKI development after ICU admission (AUC-ROC 0.839, 95% CI 0.700–0.979 and AUC-ROC 0.743, 95% CI 0.594–0.892, respectively, *P* = 0.048). Among the remaining patients that did not have hyperlactatemia (n = 83), there were no differences in the AUC-ROCs of NGAL or L-FABP for AKI prediction.

**Figure 3:**
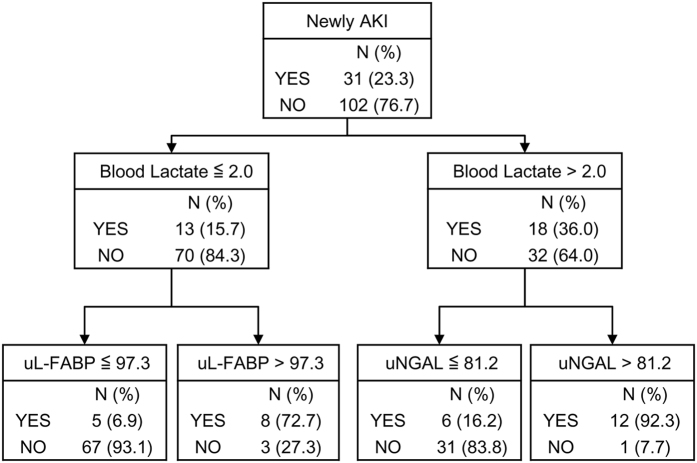
Decision tree by classification and regression tree (CART) analysis. For patients with low blood lactate levels (≤2 mmol/L), urinary L-FABP is the best splitter, with a threshold of 97.3 ng/mL. Acute kidney injury (AKI) development in patients with hyperlactatemia (>2 mmol/L) can be predicted by urinary NGAL with a threshold of 81.2 ng/mL. NGAL, neutrophil gelatinase-associated lipocalin; L-FABP, L-type fatty acid-binding protein.

We further conducted a classification and regression tree (CART) analysis to confirm that categorisation of patients according to blood lactate levels improved the predictive performance of urine NGAL and L-FABP as AKI biomarkers. Among 133 patients without a preexisting AKI diagnosis at the time of ICU admission, CART analysis showed no significant difference between their performances; the AUC-ROC of NGAL and L-FABP was 0.759 (95% CI 0.667–0.850) and 0.746 (95% CI 0.662–0830), respectively. However, CART analysis revealed that the best predictor of newly developed AKI was urinary L-FABP in patients without hyperlactatemia, i.e. those with blood lactate ≤2 mmol/L, whereas the best predictor for patients with hyperlactatemia, i.e. those with blood lactate >2 mmol/L, was urinary NGAL (Fig. 3). No remarkable improvement was observed when the decision tree was enlarged with other variables. The combined predictive ability of NGAL, L-FABP and blood lactate levels (AUC-ROC 0.847, 95% CI 0.758–0.935) was better than that of NGAL alone (*P* = 0.023) or L-FABP alone (*P* = 0.085). The significant improvement achieved with the combination model was demonstrated by category-free net reclassification index (cfNRI) and integrated discrimination index (IDI) analyses (Table 2).

### Impact of liver function on the predictive ability of NGAL and L-FABP

According to the principal component analysis, false positivity of urinary L-FABP as a predictor for AKI in patients with liver dysfunction was assumed. In the present study, liver dysfunction was detected by increases in either aspartate aminotransferase, alanine aminotransferase, or total bilirubin levels more than 1.5 times of upper normal limits that were defined as follows: 37 IU/L in males and 31 IU/L in females for aspartate aminotransferase, 40 IU/L in males and 31 IU/L in females for alanine aminotransferase and 1.2 mg/dL for total bilirubin^31^,^36^. Results showed that false positivity of L-FABP was detected only in two patients with liver dysfunction without any significant impact on the combined predictive performance of NGAL and L-FABP.

### Impact of sepsis on the predictive ability of NGAL and L-FABP

Next, we evaluated the impact of inflammation on the predictive ability as principal component analysis demonstrated simultaneous elevations of inflammatory variables and urinary NGAL, indicating significant association of inflammation with NGAL. As blood lactate level is commonly used to distinguish severe sepsis from less severe sepsis^37^,^38^, we examined whether the predictive ability would be improved by applying the decision tree (Fig. 3), which stratified patients by blood lactate levels, to septic patients. Presence of sepsis was determined according to the SCCM/ESICM/ACCP/ATS/SIS definition^39^. In septic patients (n = 40), the combined predictive performance of NGAL and L-FABP for newly developed AKI was notably improved by the decision tree, compared with NGAL alone (AUC-ROC 0.941, 95% CI 0.852–1.000 and AUC-ROC 0.750, 95% CI 0.614–0.886, respectively; *P* = 0.006).

### Impact of systemic severity on the predictive ability of NGAL and L-FABP

Finally, we examined the impact of systemic severity using the Acute Physiology and Chronic Health Evaluation (APACHE) II score^40^. Non-septic patients (n = 93) showed significantly lower APACHE II scores than septic patients (n = 40); the median APACHE II score in non-septic patients was 14. For less severe non-septic patients with APACHE II scores < 14 (n = 43), both NGAL and L-FABP showed good predictive performances for AKI (NGAL, AUC-ROC 0.983, 95% CI 0.864–0.998; L-FABP, AUC-ROC 0.950, 95% CI 0.753–0.992). Conversely, among more severe non-septic patients with APACHE II scores ≥14, the predictive performances of NGAL and L-FABP with AUC-ROC values ≤0.7 were not diagnostic for AKI. Moreover, among these more severe patients, none of the additional clinical parameters, such as blood lactate levels and liver function, improved the predictive performances of NGAL and L-FABP.

### Impact of clinical context on the predictive ability of NGAL and L-FABP

**Figure 4:**
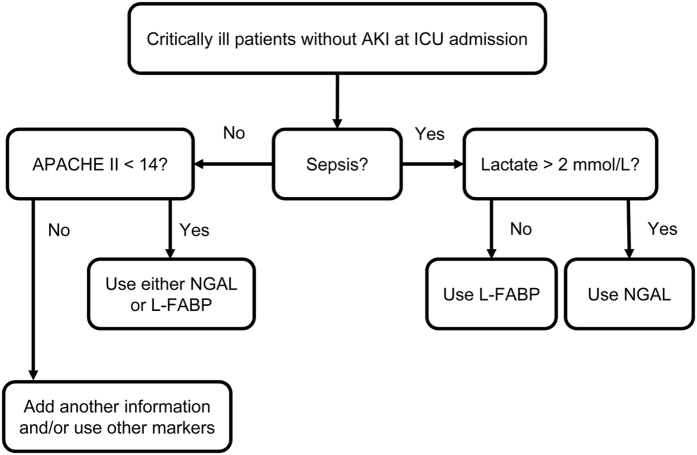
Algorithm of NGAL and L-FABP as biomarkers for AKI prediction.

Based on the results described above, we developed a new algorithm stratifying patients according to the clinical contexts (Fig. 4). This algorithm indicated that NGAL or L-FABP were good predictors of AKI in non-septic patients with APACHE II scores <14 as well as in septic patients. We compared the predictive ability of the decision tree model incorporating both NGAL and L-FABP with those of the NGAL-alone and the L-FABP-alone models in this population (n = 83). L-FABP was used for septic patients without hyperlactatemia while NGAL was used for other patients in the decision tree model. The decision tree model based on clinical context exhibited significantly better predictive ability than the models based on single AKI biomarkers (Table 3).

## Discussion

As generalisation of emerging biomarkers failed to predict and diagnose AKI with high sensitivity and specificity, their use within the clinical context is recommended^10^. We predicted that this approach with additional consideration of the potential impact of nonrenal factors on these biomarkers would improve their accuracy. The present study demonstrated that urinary NGAL and L-FABP were independent from each other by principal component analysis; NGAL showed linear correlations with inflammatory variables, whereas L-FABP correlated with the indicators of hypoperfusion and hepatic injury. Based on these findings, we propose a new algorithm for urinary NGAL and L-FABP measurement at ICU with stratification of patients by APACHE II score, presence of sepsis and blood lactate levels. Significant improvements for AKI prediction were demonstrated when NGAL and L-FABP were combined with stratification by clinical context, when non-septic patients with high APACHE II scores were excluded. Therefore, the present study demonstrated that AKI biomarker performances could be improved by a combinational approach with consideration of nonrenal conditions and systemic severity of disease.

Although NGAL and L-FABP are the most widely investigated biomarkers for renal tubular cellular injury, the mechanisms leading to their increase in plasma and urine are distinct. Tubular epithelial cells are considered to be the primary source of NGAL detected in urine; however, NGAL expression in nonrenal tissues, including neutrophils, is induced during inflammation, resulting in increased blood NGAL levels with subsequent elevations in the urine via glomerular filtration and reduced tubular reabsorption^20^. Oikonomou *et al.* reported that serum NGAL levels were strongly associated with inflammatory molecules such as C-reactive protein in patients with inflammatory bowel disease^41^. Similarly, a significant correlation between urinary NGAL levels and inflammatory markers in septic neonates was also reported^42^. Conversely, L-FABP was shown to reflect organ hypoperfusion, including that in the kidney and liver^43^,^44^. In neonates with necrotising enterocolitis, urinary L-FABP levels were shown to correlate with blood lactate levels^45^. The results of our principal component analysis were supported by these studies: Urinary NGAL correlated with markers of inflammation, whereas L-FABP showed significant linear correlations with hypoperfusion and hepatic injury markers (Fig. 2). It should be noted that serum creatinine correlated not with L-FABP but with NGAL, suggesting that urinary NGAL might be affected by glomerular filtration and renal reabsorption as described above and that L-FABP might reflect tubular epithelial cellular injury independent of glomerular filtration.

The principal component analysis provided new information indicating that specific clinical contexts might increase the predictive abilities of urinary NGAL and L-FABP. Based on these results, we were able to develop a new algorithm for predicting AKI by NGAL and L-FABP measurements in conjunction with consideration of the clinical context (Fig. 4). For septic patients, stratification using blood lactate levels aided in determination of the specific biomarker that should be measured. Sepsis was assumed to be the major cause of AKI, given that up to 45% of the patients with AKI were septic in the present study. Blood lactate reflects tissue hypoperfusion frequently observed in severe sepsis. Conversely, blood lactate levels can be elevated by mechanisms other than hypoperfusion, including mitochondrial dysfunction and pyruvate dehydrogenase deficiency as well. Based on advances in the understanding of these mechanisms, blood lactate level is increasingly used for defining septic shock, the most severe form of sepsis, in the latest definition of sepsis^46^. Thus, we used the same cut-off value of 2.0 mmol/L for septic patient stratifications in the new algorithm. While several previous studies suggested that L-FABP might be useful for detection of AKI caused by septic shock^23^,^47^, L-FABP might fail to perform as a better predictor of AKI compared with NGAL in septic patients with hyperlactatemia, as several different mechanisms other than renal injury might increase both lactate and L-FABP. One such potentially confounding mechanism is the impact of altered liver function on blood lactate levels. Decreased lactate metabolism in the injured liver, which could not be detected with aminotransferases and bilirubin, might increase blood lactate levels and result in false positive results with L-FABP as a biomarker for AKI.

Our new algorithm revealed that the predictive performances of NGAL and L-FABP were not good in non-septic patients with high-APACHE II scores, which was consistent with a previous study, which reported that NGAL was less discriminative in non-septic patients with relatively high APACHE II scores^18^. In addition to sepsis, several systemic diseases, including acute respiratory distress syndrome (ARDS) and acute heart failure, can also induce AKI^48^,^49^. In ARDS patients, urine interleukin 18 can be used for early detection of AKI^50^. Ronco *et al.* reported that the combination of B-type natriuretic peptide and NGAL was useful for diagnosis and prevention of AKI in patients with acute decompensated heart failure^49^. These studies indicated that more information on systemic conditions and incorporation of additional biomarkers are expected to improve predictive performances of AKI biomarkers in complicated disease states. Further evaluation is necessary to confirm our findings.

This study has several limitations. First, the number of patients included in this single centre study was limited. However, this study demonstrated that a new approach using principal component analysis and decision tree algorithms could clarify the distinct characteristics of two AKI biomarkers, NGAL and L-FABP. Future multicentre studies with larger cohorts should be conducted to reassess our strategy and findings. Second, we did not use the latest definition of sepsis in stratification^46^. Recent changes introduced to define sepsis, which should not dramatically alter its clinical diagnosis, might influence the utility of our algorithm.

In conclusion, this study emphasised that clinical contexts, such as nonrenal conditions and systemic disease severity, should be considered in utilisation of new biomarkers for AKI prediction. We successfully developed a new algorithm that improved the predictive performance of urinary NGAL and L-FABP by stratifying AKI patients using APACHE II score, presence of sepsis and blood lactate levels.

## Methods

This was a prospective observational study. The University of Tokyo Institutional Review Board approved all study procedures and materials. Informed consent was obtained from all patients or their guardians. All procedures were performed in accordance with the directives of the Declaration of Helsinki.

### Participants and data collection

Patients aged 18 years or older who were admitted to the ICU of the University of Tokyo Hospital were considered for study inclusion. The exclusion criterion was the presence of end-stage renal disease, i.e. chronic dialysis and/or kidney transplantation.

The following clinical variables included in the medical records were evaluated: age, sex, APACHE II score^40^, reason for ICU admission, presence of sepsis diagnosed according to the International SCCM/ESICM/ACCP/ATS/SIS definition^39^, past history of hypertension and diabetes mellitus, baseline serum creatinine and baseline CKD status. AKI was determined by changes in serum creatinine levels according to the Kidney Disease Improving Global Outcomes (KDIGO) criteria^51^ and was defined as an increase in serum creatinine level by 0.3 mg/dL within 48 h or at least 1.5 times over baseline levels. Baseline serum creatinine was defined as the minimum value among all outpatient values within the last 6 months prior to hospital admission, inpatient value before ICU admission and last value before hospital discharge, as previously described^52^. In patients for whom no creatinine values within the last 6 months prior to ICU admission were available, the baseline was defined as the minimum value among the creatinine values immediately before hospital discharge and the estimated creatinine value using the Modification of Diet in Renal Disease equation for Japan^53^ for the lower end of the reference range (i.e. 75 mL/min/1.73 m^2^), as suggested by the KDIGO guidelines. CKD was defined as abnormalities in kidney structure or function that were present for >3 months, with adverse health implications^54^. All blood and urine samples were collected and analysed immediately at the time of ICU admission. Measured variables included C-reactive protein, white blood cell count, blood lactate, aspartate aminotransferase, alanine aminotransferase, total bilirubin, serum creatinine and urinary NGAL and L-FABP levels. Urinary L-FABP and NGAL levels were measured by enzyme-linked immunosorbent assay (Human L-FABP Assay Kit; CMIC, Tokyo, Japan) and chemiluminescent microparticle immunoassay (ARCHITECT Urine NGAL; Abbot, Abbot Park, IL, USA). All other measurements were conducted at the University of Tokyo Hospital Clinical Laboratory.

### Evaluation of predictive performance of biomarkers for AKI development

The predictive ability of each biomarker was assessed using multivariable logistic regression analysis and evaluated by AUC-ROC. CART analyses were also conducted to estimate predictive ability^55^ using age, APACHE II score, presence of sepsis, C-reactive protein, white blood cell count, blood lactate, aspartate aminotransferase, alanine aminotransferase, total bilirubin and urinary NGAL and L-FABP levels as variables. Statistical differences in AUC-ROCs were evaluated using the method described by Delong *et al.*^56^. To assess the incremental performance of a predictive model, cfNRI and IDI were calculated^57^. cfNRI and IDI were computed using the PredictABEL R package^58^.

### Statistical analysis

Continuous variables were described as medians with interquartile range and compared using Student’s *t* test or Wilcoxon rank-sum test where appropriate. Categorical variables were described as percentages and compared using the chi-square test or Fisher’s exact test. *P* values of <0.05 were considered statistically significant. CART analyses were performed with Salford Predictive Modeler v7.0 (Salford Systems, San Diego, CA, USA), and all other analyses were performed using R v3.1.1 (R Project for Statistical Computing, Vienna, Austria; http://www.R-project.org).

## Additional Information

**How to cite this article**: Asada, T. *et al.* Impact of clinical context on acute kidney injury biomarker performances: differences between neutrophil gelatinase-associated lipocalin and L-type fatty acid-binding protein. *Sci. Rep.* **6**, 33077; doi: 10.1038/srep33077 (2016).

## Figures and Tables

**Table 1:** Baseline characteristics of patients without acute kidney injury (AKI) at the time of intensive care unit admission.

	New AKI patients (n = 31)	Patients with no AKI (n = 102)	*P* value
Age (year)	63 (48–74)	63 (51–73)	0.74
Male, no. (%)	21 (68)	59 (58)	0.40
Surgical, no. (%)	8 (26)	49 (48)	0.04
Sepsis, no. (%)	14 (45)	26 (25)	0.04
respiratory, no. (%)	6 (43)	15 (60)	
abdominal, no. (%)	1 (7)	2 (8)	
urinary, no. (%)	1 (7)	2 (8)	
others, no. (%)	6 (43)	6 (24)	
APACHE II score	19 (15–26)	15 (11–19)	<0.001
Hypertension, no. (%)	17 (55)	42 (41)	0.22
Diabetes Mellitus, no. (%)	10 (32)	16 (16)	0.07
Baseline creatinine (mg/dL)	0.91 (0.55–1.63)	0.63 (0.51–0.78)	<0.001
Baseline CKD, no. (%)	10 (32)	7 (6.9)	<0.001
C-reactive protein (mg/dL)	2.7 (0.3–9.5)	0.9 (0.1–5.6)	0.08
White blood cell count (×10^3^/μL)	11.6 (5.2–16.0)	11.8 (7.7–14.6)	0.63
Blood lactate (mmol/L)	2.3 (1.2–4.3)	1.6 (1.1–2.3)	0.03
Aspartate aminotransferase (IU/L)	36 (24–57)	29(20–38)	0.11
Alanine aminotransferase (IU/L)	18 (13–50)	17 (11–26)	0.06
Total bilirubin (mg/dL)	0.7 (0.4–1.1)	0.7 (0.5–1.1)	0.91
Serum creatinine (mg/dL)	1.27 (0.8–2.1)	0.65 (0.53–0.83)	<0.001
Urine NGAL (ng/mL)	123.2 (19.0–699.2)	12.5 (4.9–34.5)	<0.001
Urine L-FABP (ng/mL)	88.1 (29.4–211.9)	9.2 (3.5–36.0)	<0.001

APACHE, Acute Physiology and Chronic Health Evaluation; CKD, chronic kidney disease; NGAL, neutrophil gelatinase-associated lipocalin; L-FABP, L-type fatty acid-binding protein.

**Table 2:** Predictive ability of combination model with CART analysis.

	vs. NGAL only	vs. L-FABP only
NRI		
% Events to higher risk	65.7	74.5
% Events to lower risk	34.3	25.5
% Non-events to higher risk	16.1	25.8
% Non-events to lower risk	83.9	74.2
Total cfNRI	0.99*	0.97*
(95% CI)	(0.67–1.3)	(0.62–1.3)
Total IDI	0.205*	0.285*
(95% CI)	(0.108–0.303)	(0.177–0.393)

^*^P < 0.001

CART, classification and regression tree; CI, confidence interval; cfNRI, category-free net reclassification index; IDI, integrated discrimination index; NGAL, neutrophil gelatinase-associated lipocalin; L-FABP, L-type fatty acid-binding protein.

**Table 3:** Comparison of acute kidney injury (AKI) predictive ability.

	NGAL alone	L-FABP alone	Decision Tree Analysis
AUC-ROC (95% CI)	0.858 (0.741–0.927)	0.837 (0.697–0.920)	0.940 (0.793–0.985)
NGAL alone	—	*P* = 0.67	*P* = 0.03
L-FABP alone	—	—	*P* = 0.007

AUC-ROC, area under the receiver operating characteristic curve; CI, confidence interval; NGAL, neutrophil gelatinase-associated lipocalin; L-FABP, L-type fatty acid-binding protein.
